# Temporal differences in gamma-hydroxybutyrate overdoses involving injecting drug users versus recreational drug users in Helsinki: a retrospective study

**DOI:** 10.1186/1757-7241-20-7

**Published:** 2012-02-01

**Authors:** James J Boyd, Markku J Kuisma, Tarja T Randell

**Affiliations:** 1 Helsinki Emergency Medical Service, Helsinki University Central Hospital, Helsinki, Finland; 2 Department of Anaesthesiology and Intensive Care Medicine, Helsinki University Central Hospital, Helsinki, Finland

**Keywords:** Gamma-hydroxybutyric acid, Emergency Medical Services, overdose

## Abstract

**Background:**

Gamma-hydroxybutyrate (GHB) and gamma-butyrolactone (GBL) have been profiled as 'party drugs' used mainly at dance parties and in nightclubs on weekend nights. The purpose of this study was to examine the frequency of injecting drug use among GHB/GBL overdose patients and whether there are temporal differences in the occurrence of GHB/GBL overdoses of injecting drug and recreational drug users.

**Methods:**

In this retrospective study, the ambulance and hospital records of suspected GHB- and GBL overdose patients treated by the Helsinki Emergency Medical Service from January 1^st ^2006 to December 31^st ^2007 were reviewed. According to the temporal occurrence of the overdose, patients were divided in two groups. In group A, the overdose occurred on a Friday-Saturday or Saturday-Sunday night between 11 pm-6 am. Group B consisted of overdoses occurring on outside this time frame.

**Results:**

Group A consisted of 39 patient contacts and the remaining 61 patient contacts were in group B. There were statistically significant differences between the two groups in (group A vs. B, respectively): history of injecting drug abuse (33% vs. 59%, p = 0.012), reported polydrug and ethanol use (80% vs. 62%, p = 0.028), the location where the patients were encountered (private or public indoors or outdoors, 10%, 41%, 41% vs. 25%, 18%, 53%, p = 0.019) and how the knowledge of GHB/GBL use was obtained (reported by patient/bystanders or clinical suspicion, 72%, 28% vs. 85%, 10%, p = 0.023). Practically all (99%) patients were transported to emergency department after prehospital care.

**Conclusion:**

There appears to be at least two distinct groups of GHB/GBL users. Injecting drug users represent the majority of GHB/GBL overdose patients outside weekend nights.

## Background

Gamma-hydroxybutyrate (GHB) and gamma-butyrolactone (GBL) have been profiled as 'party drugs' used mainly at dance parties, in nightclubs or other similar settings [1-4]. They have also been used by bodybuilders and to facilitate sexual assault [3]. The effects of GHB/GBL range from euphoria, disinhibition and agitation to reduced level of consciousness, respiratory depression and death [3].

The Helsinki Emergency Medical Services (EMS) encounters GHB/GBL overdose patients outside the more conventional setting of weekend nights. Also, many of the patients have a history of injecting drug use. The aims of this study were to examine the proportion of patients with GHB/GBL overdose presenting to the EMS outside weekend nights and the frequency of injecting drug use among GHB/GBL overdose patients.

## Methods

The study plan and hypothesis were retrospective, although data were collected prospectively for quality assurance purposes. Helsinki, the capital city of Finland had a population of 560,000 during the study period. Helsinki consist of both urban and semi-urban areas.

The Helsinki EMS responds to all urgent calls in the city and is composed of a three tiered system. The first tier consisting of emergency medical technician (EMT), trained firemen manning the ambulances and fire engines used as first responding units. The second tier consists of three advanced life support (ALS) units manned by paramedics and one paramedic supervisor unit. The paramedics are licensed to administer intravenous drugs after physician consultation or by following written standing orders. The third tier is made up of a mobile intensive care unit (MICU) staffed by two EMT-firemen and an emergency physician. One emergency physician is on-call at a time. The on-call physician is responsible for consulting both EMS personnel and private ambulance company personnel and participates in the care of high-risk patients personally.

Institutional approval was obtained. The EMS records of patients with serious presumed GHB or GBL overdoses were collected prospectively on-line by the on-call emergency physician and reviewed by one of the authors (JB) from January 1^st ^2006 to December 31^st ^2007. All applicable cases from this time period were included in the study. We defined a presumed GHB/GBL overdose as any patient with either reported use of GHB/GBL or clinical findings suggesting GHB/GBL overdose and GHB/GBL found on person. Clinical findings suggesting a GHB/GBL overdose were considered to be: a reduced level of consciousness with periods of agitation, respiratory depression and bradycardia [3,4]. The recognition of a GHB overdose by clinical signs and symptoms has been previously shown to be reliable with a sensitivity of 97% and specificity of 91% [2]. Clinical suspicion of GHB intoxication has been used as an inclusion criteria also in a previous study [5]. An overdose was considered to be serious if the patient had a reduced level of consciousness and/or respiratory depression.

The patients were then divided in to two groups according to the time of call to the emergency dispatching centre. In group A, the call was made on Friday-Saturday or Saturday-Sunday between 11 p.m. and 6 a.m. In group B, the call was made outside this time frame. In a previous study on patients attending an emergency department (ED) after GHB ingestion, the majority (54%) of the patients attended the ED between 12 midnight and 8 a.m. [5]. In the present study, the time of the call to the emergency dispatching centre was used instead of ED attendance time. As this time would precede the time of attendance to an ED, the timeframe of 11 p.m. and 6 a.m. was used. Especially Friday and Saturday nights were described as "party" nights, a potential time for recreational drug use, in a previous study on club drug use (e.g. GHB) [6].

For each patient, we recorded the date and time of the call to the emergency dispatching centre, age, sex, location of the overdose, previous substance abuse, agents involved in current overdose, level of consciousness and respiration on arrival of the first responding unit and after treatment, treatment provided by the EMS personnel and disposition of the patient. When considering the location of the overdose, a private residence was marked as 'private, indoors'. Bars, restaurants, discos, night-clubs and metro-stations were marked as 'public, indoors' and streets, parks or vehicles were marked as 'outdoors'. When possible, alcohol breath analyzers were used in the prehospital phase (Dräger alcotest 6510, Drägerwerk AG & Co. KGaA 23542 Lübeck, Germany). Hospital records were reviewed for conformation of the diagnosis of GHB/GBL overdose, level of care required in the emergency department (ED), disposition and previous substance abuse.

Chi-square and Mann-Whitney tests were used when appropriate. p-value less than 0.05 was considered statistically significant. Statistics were calculated using SPSS statistical software (SPSS for Windows version 16.0, SPSS Inc., Chicago, IL).

## Results

Helsinki EMS ambulances were dispatched to 40,993 urgent calls in 2006. Out of these, 2952 were coded as intoxications or overdoses. In 2007, the respective figures were 46,102 and 3089. In addition to GHB and GBL overdoses, the Helsinki EMS treated 24 and 26 non-fatal presumed opioid overdoses in 2006 and 2007, respectively. Two patients had used GBL and alcohol with crushed, dissolved and injected buprenorphine tablets. They had the clinical picture of an opioid overdose and responded to naloxone.

During the study period there were 100 GHB/GBL overdoses involving 90 patients. Eight patients were treated more than once. Forty one patients had a history of injecting drug use according to the hospital records and thirteen had also previous GHB/GBL use. The history of injecting drug use included amphetamine (n = 19), buprenorphine (n = 10), heroin (n = 3) and unspecified injected drugs (n = 19). In addition, alcohol and benzodiazepines were widely used. During the study period, EMS treated four of these patients twice and two patients three times due to GHB/GBL overdose. Forty nine patients did not have previous injecting drug use recorded, but seven had previous GHB/GBL use according to hospital records. Two of these patients were treated twice by EMS due to GHB/GBL overdose during the study period.

The annual occurrence of GHB/GBL overdoses treated by EMS increased from 32 patient contacts in 2006 to 68 patient contacts in 2007. Among these, GHB/GBL overdoses involving injecting drug users increased from 18 to 31 patient contacts during the same time period.

**Figure 1:**
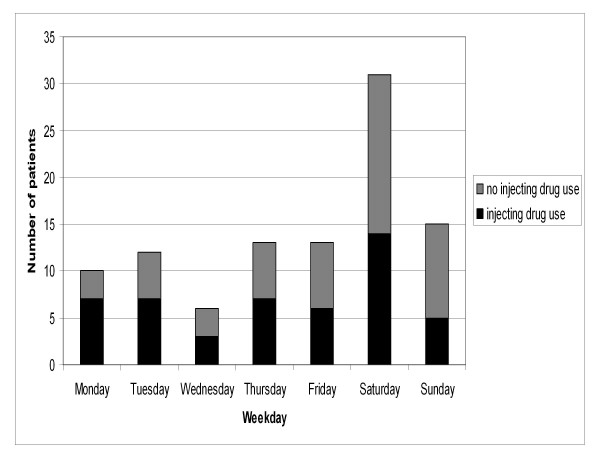
**Distribution of overdoses according to the weekday**. Patients with serious GHB and GBL overdose treated by Helsinki EMS in 2006-2007. EMS: Emergency Medical Service, GHB: gammahydroxybutyrate, GBL: gammabutyrolactone.

**Figure 2:**
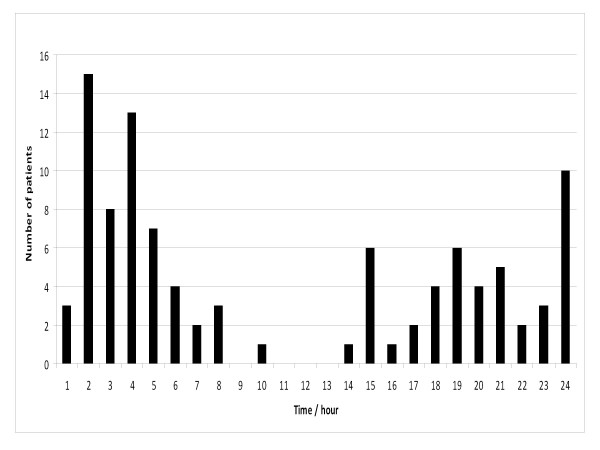
**Distribution of overdoses according to the time of day**. Patients with serious GHB and GBL overdose treated by Helsinki Emergency Medical Services in 2006-2007. EMS: Emergency Medical Service, GHB: gammahydroxybutyrate, GBL: gammabutyrolactone.

The temporal distribution of all the patient contacts according to the weekday and the time of day are presented in Figures 1 and 2, respectively. The call to the emergency dispatching centre was placed on Friday-Saturday or Saturday-Sunday night between 11 pm-6 am in thirty nine patient contacts (Group A). The majority, sixty one, of the patient contacts were outside this time frame (Group B). Almost all the patients were encountered either from the city centre or an area North-east of the city centre (Kallio) or in their immediate vicinity.

**Figure 3:**
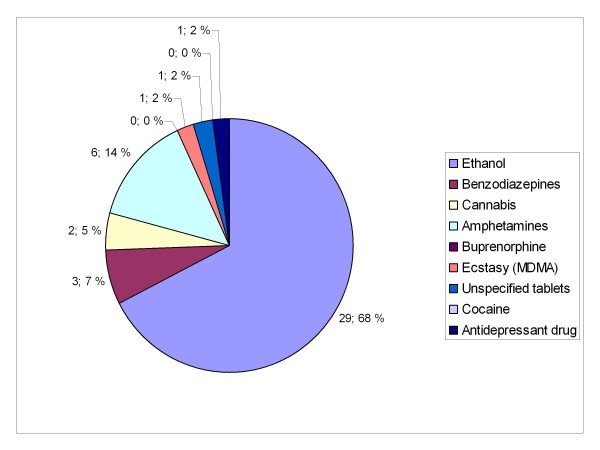
**Concomitantly used substances in group A**. Patients with serious GHB and GBL overdose treated by Helsinki EMS in 2006-2007. Call to emergency dispatching centre made on Friday-Saturday or Saturday-Sunday night between 11 pm and 6 am. EMS: Emergency Medical Service, MDMA: 3,4-methylenedioxymethamphetamine.

**Figure 4:**
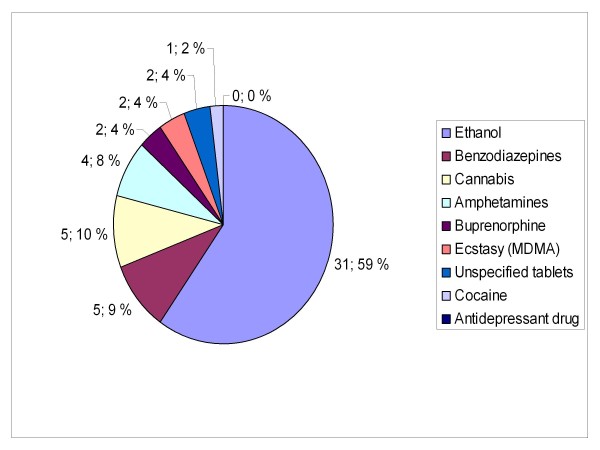
**Concomitantly used substances in group B**. Patients with serious GHB and GBL overdose treated by Helsinki EMS in 2006-2007. Group B: call to emergency dispatching centre made outside the time frame of weekend nights. EMS: Emergency Medical Service, MDMA: 3, 4-methylenedioxymethamphetamine.

Patient characteristics are presented in table 1 and concomitantly used drugs and medications are presented in Figures 3 and 4. There were statistically significant differences between the two groups in history of injecting drug use (p = 0.012) and reported concomitant use of other drugs, medications and/or ethanol; 31 (80%) patients in group A versus 38 (62%) patients in group B, p = 0.028.

**Table 1:** Patient characteristics and prehospital airway management.

	Group A	Group B	p-value
n	39 (39%)	61 (61%)	
Sex (male)	19 (49%)	35 (57%)	0.397
Age*	24 (22;27)	25 (23;29)	0.134
History of injecting drug use	13 (33%)	36 (59%)	0.012
Intubated by EMS on scene	8 (21%)	14 (23%)	0.774

Patients with serious GHB and GBL overdose treated by Helsinki EMS in 2006-2007.

*Median and 25- and 75-percentiles.

Group A: call to emergency dispatching centre made on Friday-Saturday or Saturday-Sunday night between 11 pm and 6 am. Group B: call to emergency dispatching centre made at other times.

GHB: gammahydroxybutyrate, GBL: gammabutyrolactone, EMS: Emergency Medical Service.

No statistically significant differences were noted in the EMS use of prehospital naloxone and flumazenil between the two groups (p = 0.083). In group A, naloxone was not administered, but flumazenil was administered to six patients. In group B, both naloxone and flumazenil were administered to one patient and naloxone or flumazenil alone was administered to five patients, respectively. There was no difference in intubation rate between the two groups (Table 1).

**Figure 5:**
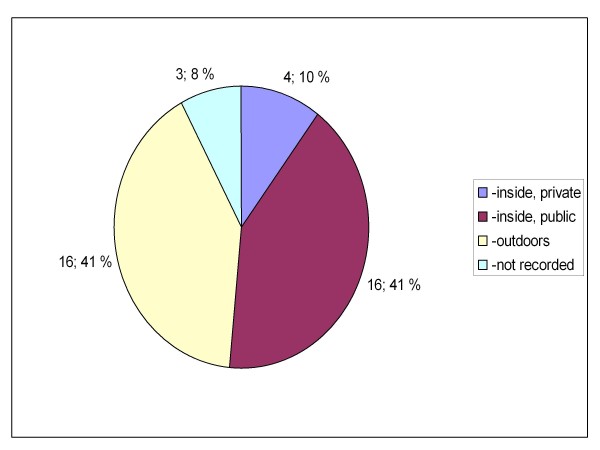
**Location of overdose in group A**. Patients with serious GHB and GBL overdose treated by Helsinki EMS in 2006-2007. Group A: call to emergency dispatching centre made on Friday-Saturday or Saturday-Sunday night between 11 pm and 6 am. EMS: Emergency Medical Service, GHB: gammahydroxybutyrate, GBL: gammabutyrolactone.

**Figure 6:**
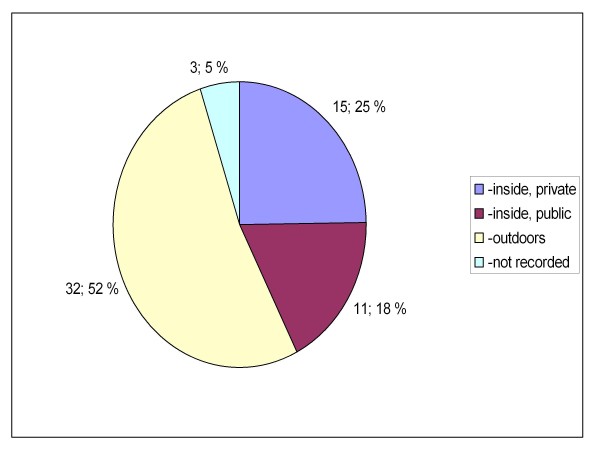
**Location of overdose in group B**. Patients with serious GHB and GBL overdose treated by Helsinki EMS in 2006-2007. Group B: call to emergency dispatching centre made outside the time frame of weekend nights. EMS: Emergency Medical Service, GHB: gammahydroxybutyrate, GBL: gammabutyrolactone.

**Figure 7:**
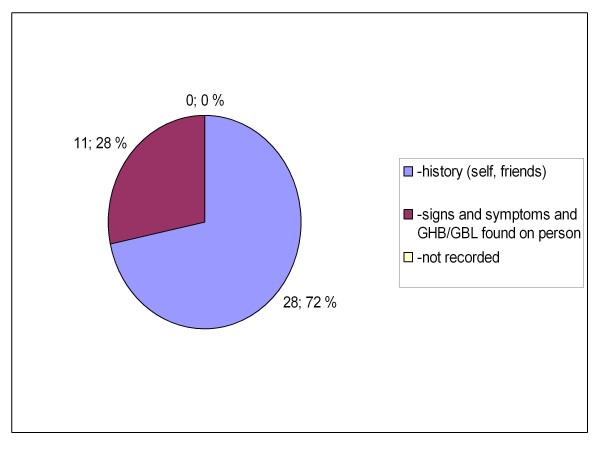
**Information on GHB or GBL use in group A**. Patients with serious GHB and GBL overdose treated by Helsinki EMS in 2006-2007. Group A: call to emergency dispatching centre made on Friday-Saturday or Saturday-Sunday night between 11 pm and 6 am. EMS: Emergency Medical Service, GHB: gammahydroxybutyrate, GBL: gammabutyrolactone.

**Figure 8:**
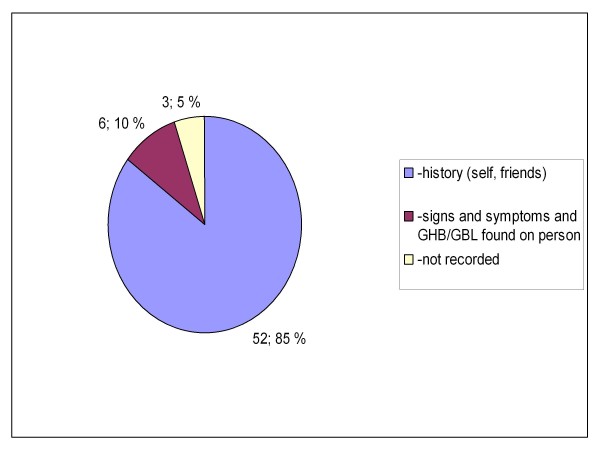
**Information on GHB or GBL use in group B**. Patients with serious GHB and GBL overdose treated by Helsinki EMS in 2006-2007. Group B: call to emergency dispatching centre made outside the time frame of weekend nights. EMS: Emergency Medical Service, GHB: gammahydroxybutyrate, GBL: gammabutyrolactone.

**Figure 9:**
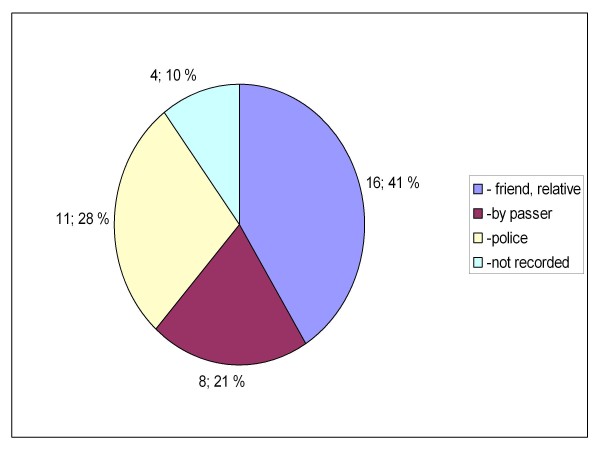
**Caller to emergency dispatching centre in group A**. Patients with serious GHB and GBL overdose treated by Helsinki EMS in 2006-2007. Group A: call to emergency dispatching centre made on Friday-Saturday or Saturday-Sunday night between 11 pm and 6 am. EMS: Emergency Medical Service, GHB: gammahydroxybutyrate, GBL: gammabutyrolactone.

**Figure 10:**
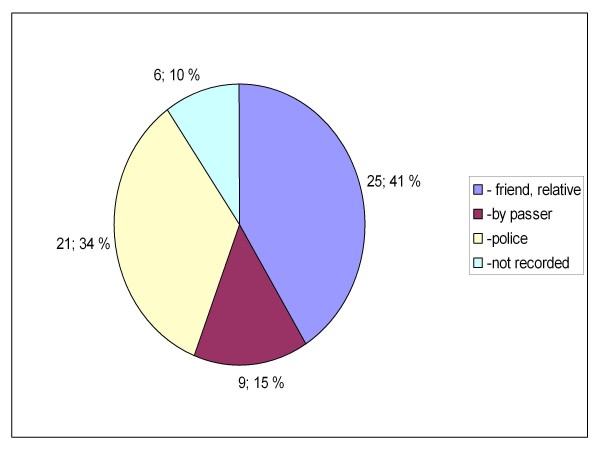
**Caller to emergency dispatching centre in group B**. Patients with serious GHB and GBL overdose treated by Helsinki EMS in 2006-2007. Group B: call to emergency dispatching centre made outside the time frame of weekend nights. EMS: Emergency Medical Service, GHB: gammahydroxybutyrate, GBL: gammabutyrolactone.

Between the two groups, there were also statistically significant differences in the location where the patients were encountered (private or public indoors or outdoors, p = 0.019, Figures 5 and 6) and how the knowledge of GHB/GBL use was obtained (self reported, reported by bystanders or clinical suspicion, p = 0.023 (Figures 7 and 8). There was no difference between the two groups in the caller to the emergency dispatching centre (p = 0.687, Figures 9 and 10). Practically all (99%) patients were transported after prehospital care to one of the three local EDs.

## Discussion

In our investigation, the majority (60%) of GHB/GBL overdoses occurred from 11 p.m. to 6 a.m. and 55% occurred during weekend from Friday evening to Sunday (Figure 1 and 2). Similar findings have been reported previously [5,7], with up to 92% of the patients arriving to an ED between Friday 5 p.m. and Monday 8 a.m. [8]. These findings are probably related to the role of GHB/GBL as a recreational drug [1-3]. Among injecting heroin users, similar concentration on weekends has been found in overdoses [9], but in comparison to GHB users most the heroin overdoses occurred in the early evening or before midnight [9,10]. Patients were predominately encountered in areas were the city's night life is concentrated (city centre, Kallio) or where substance abuse is popular (Kallio).

In the present study, 54% of all the patients and 49% of patients in group A were male. This is lower than in some of the previous studies, where 60-93% of the GHB-related medical emergency patients were male [1,4,5,8,11]. The higher portion of males could be related to the high popularity of GHB/GBL amongst homosexual men in the study populations [4,6,11]. Yet, some studies on another recreational drug, MDMA ('Ecstasy' or 3,4-methylenedioxymethamphetamine), have failed to show gender differences in use [12,13]. Females also suffer from acute adverse effects of drug abuse more often as they usually dose at similar rates as men while being of smaller size and having different physiology [14]. Also, among suspected exposures to GHB and congener drugs reported to the California Poison Control System the proportion of women increased from 38% in 1999 to 60% in 2003 [15]. However, this increase could be partly explained by the proportional increase of 'malicious' events, such as sexual assaults, where the majority of victims were female [15].

There appears to be at least two distinct groups of GHB/GBL users. The majority of the patients in group A used GHB/GBL with alcohol and overdosed during weekend nights in night-clubs and discos (public places). On the other hand, the majority of the patients in group B had a history of injecting drug use, overdosed in the middle of the week or in the early evenings and were encountered more often in private residences and outdoors (Tables 1, Figures 5 and 6). Injecting drug users also had more often repeated GHB/GBL overdoses in comparison to non injecting drug users. Similar differences in the relative location of the overdose have been noted when comparing GHB- and heroin overdose patients previously [1].

Temporal differences in GHB/GBL overdoses involving injecting drug users are also seen in more recent data, although less than in 2006-2007. Between 1.1.2009 and 31.12.2010 Helsinki EMS treated 58 GHB/GBL overdose patients. Of them, 25 (43%) had a history of injecting drug use. During weekend nights, 36% of the GHB/GBL overdose patients had a history of injecting drug use, while 45% had a similar history if encountered outside this time frame (James Boyd: Personal communication). These findings could reflect decreased use of GHB/GBL by injecting drug users. This is supported by a recent study on disadvantaged drug users in Helsinki, where 6% of the interviewed reported use of GHB/GBL within the past month, while 22% reported use over 12 months previously [16]. To our knowledge, the occurrence of GHB/GBL overdoses that involve injecting drug users has not been previously reported.

Polysubstance use is common with GHB use [2-5,8,11]. Polydrug use, as such, was less often reported in group B (Figures 3 and 4). This could be due to the concomitant use of other, more illegal substances, by injecting drug users and thus unwillingness to report them. Also, although there were no statistically significant differences, naloxone and flumazenil were administered by EMS more often in this group.

Users of amphetamine, buprenorphine and heroin typically inject their drugs in Finland [16,17]. Since the beginning of the century there has been a remarkable decline in heroin related overdoses in Finland [18], while buprenorphine abuse has increased [17,19]. In 2007, the number of GHB/GBL overdose patient contacts, with a history of injecting drug use, surpassed that of opioid overdose patient contacts (31 vs. 26, respectively) in our study population.

As polydrug use is very common among injecting drug users in Finland to begin with [16,17] and GHB/GBL are relatively easy to obtain and cheap (a 50 ml bottle costs 5-6 €) to our knowledge, it is easy to see the potential of it as a drug of abuse also among injecting drug users. Almost one third of the patients with a history of injecting drug abuse also had a history of previous GHB/GBL use. This could implicate chronic use of GHB/GBL among this group of patients.

The clinical implications are that a GHB/GBL overdose patient encountered during weekdays has more probably a history of injecting drug use with all the related medical and social problems, such as a higher risk for infectious diseases [20,21] and homelessness [21]. Also, the possibility of a GHB/GBL overdose should be kept in mind when treating injecting drug users with a reduced level of consciousness and respiratory depression, even when evidence of currently administered injectable substances is present.

### Study limitations

The patients in this study do not represent all the patients with GHB/GBL related problems treated by the Helsinki EMS. Probably both the non severe cases and some of the unconscious patients were missed due to the lack of knowledge on GHB/GBL involvement. In addition, injecting drug users are unlikely to call for help when an overdose occurs [22], so at least part of their GHB/GBL related overdoses went unnoticed by EMS. When considering the temporal distribution, it should be noted that festivities (such as New Year's Eve, Christmas, 1^st ^of May, Midsummer) were not considered.

There was no toxicological confirmation on GHB/GBL use. The GHB/GBL use was, however, mainly reported by the overdose victims themselves or by bystanders. Only in a minority of cases clinical suspicion with liquid suspected to be GHB or GBL found on person was used as a criteria for inclusion (Figures 7 and 8). The concomitant use of other substances was self reported or reported by bystanders, except for ethanol (alcohol breath analyzers were routinely used).

## Conclusion

There appears to be at least two distinct groups of GHB/GBL users. Recreational drug users use GHB/GBL with alcohol and overdose during weekend nights while clubbing. Patients with a history of injecting use of amphetamines, heroin and/or buprenorphine represent the majority of GHB/GBL overdose patients outside weekend nights.

## Competing interests

The authors declare that they have no competing interests.

## Authors' contributions

All authors participated in devising the study plan and writing the manuscript. JB was also responsible of document retrieval and reviewing and data analysis. Finally all authors have read and approved the final manuscript

## References

[B1] Dietze PM Cvetkovski S Barratt MJ Clemens S Patterns and incidence of gamma-hydroxybutyrate (GHB)-related ambulance attendances in Melbourne, Victoria Med J Aust 2008 188 709 711 10.5694/j.1326-5377.2008.tb01851.x 18558893

[B2] West E Cameron P O'Reilly G Drummer OH Bystrzycki A Accuracy of current clinical diagnosis in recreational drug-related attendance to the emergency department Emerg Med Australas 2008 20 333 338 10.1111/j.1742-6723.2008.01110.x 18782207

[B3] Snead OC Gibson KM Gamma-hydroxybutyric acid N Eng J Med 2005 352 2721 2732 10.1056/NEJMra044047 15987921

[B4] Li J Stokes SA Woeckener A A tale of novel intoxication: Seven cases of gamma-hydroxybutyric acid overdose Ann Emerg Med 1998 31 723 728 10.1016/s0196-0644(98)70231-8 9624312

[B5] Munir VL Hutton JE Harney JP Buykx P Weiland TJ Dent AW Gamma-hydroxybutyrate: A 30 month emergency department review Emerg Med Australas 2008 20 521 530 10.1111/j.1742-6723.2008.01140.x 19125832

[B6] Kelly BC Parsons JT Wells BE Prevalence and predictors of club drug use among club-going young adults in New York City J Urban Health 2006 83 884 895 10.1007/s11524-006-9057-2 PMC2438587 16937088

[B7] Liechti ME Kunz I Greminger P Speich R Kupferschmidt H Clinical features of gamma-hydroxybutyrate and gamma-butyrolactone toxicity and concomitant drug and alcohol use Drug Alcohol Depend 2006 81 323 326 10.1016/j.drugalcdep.2005.07.010 16143455

[B8] Miro O Nogue S Espinosa G To-Figueras J Sanchez M Trends in illicit drug emergencies: The emerging role of gamma-hydroxybutyrate J Toxicol Clin Toxicol 2002 40 129 135 10.1081/clt-120004400 12126184

[B9] Ruttenber AJ Luke JL Heroin-related deaths: New epidemiologic insights Science 1984 226 14 20 10.1126/science.6474188 6474188

[B10] Manfredini R Gallerani M Calo G Pasin M Govoni M Fersini C Emergency admission of opioid abusers for overdose: A chronobiological study of enhanced risk Ann Emerg Med 1994 24 615 618 10.1016/s0196-0644(94)70270-5 8092587

[B11] Wood DM Warren-Gash C Ashraf T Greene SL Shather Z Trivedy C Clarke S Ramsey J Holt DW Dargan PI Medical and legal confusion surrounding gamma-hydroxybutyrate (GHB) and its precursors gamma-butyrolactone (GBL) and 1,4-butanediol (1, 4BD) Q J Med 2008 101 23 29 10.1093/qjmed/hcm117 18203723

[B12] Boyd CJ McCabe SE d'Arcy H Ecstasy use among collage undergraduates: Gender, race and sexual identity J Subst Abuse Treat 2003 24 209 215 10.1016/s0740-5472(03)00025-4 12810141

[B13] Boys A Marsden J Griffiths P Fountain J Stillwell G Strang J Substance use among young people: The relationship between perceived functions and intentions Addiction 1999 94 1043 1050 10.1046/j.1360-0443.1999.94710439.x 10707442

[B14] Liechti ME Gamma A Vollenweider FX Gender differences in the subjective effects of MDMA Psychopharmacol 2001 154 161 168 10.1007/s002130000648 11314678

[B15] Anderson IB Kim SY Dyer JE Burkhardt CB Iknoian JC Walsh MJ Blanc PD Trends in γ-Hydroxybutyrate (GHB) and related drug intoxication: 1999 to 2003 Ann Emerg Med 2006 47 177 183 10.1016/j.annemergmed.2005.10.012 PMC2246009 16431231

[B16] Tammi T Pitkänen T Perälä J Stadin nistit- huono-osaisten helsinkiläisten huumeidenkäyttäjien päihteet sekä niiden käyttötavat ja hankinta Yhteiskuntapolitiikka 2011 76 45 54 (abstract in English)

[B17] European monitoring centre for drugs and drug addiction (EMCDDA) 2011 http://www.emcdda.europa.eu/publications/country-overviews/fi Situation summary: Problem drug use. Last accessed July 3^rd^

[B18] European monitoring centre for drugs and drug addiction (EMCDDA) 2011 http://www.emcdda.europa.eu/publications/country-overviews/fi. Situation summary: Drug related deaths. Last accessed July 3^rd^

[B19] Alho H Sinclair D Vuori E Holopainen A Abuse liability of buprenorphine-naloxone tablets in untreated IV drug users Drug Alcohol Depend 2007 88 75 78 10.1016/j.drugalcdep.2006.09.012 17055191

[B20] Gordon RJ Lowy FD Bacterial infections in drug users N Eng Med J 2005 353 1945 1954 10.1056/NEJMra042823 16267325

[B21] Metraux S Metzger DS Homelessness and HIV risk behaviors among injection drug users J Urban Health 2004 81 618 629 10.1093/jurban/jth145 PMC3455922 15466843

[B22] Darke S Ross J Hall W Overdose among heroin users in Sydney, Australia: II Responses to overdose Addiction 1996 91 413 417 10.1046/j.1360-0443.1996.91341310.x 8867203

